# Effects of ice-slurry and carbohydrate on exercise in the heat

**DOI:** 10.1186/2046-7648-4-S1-A123

**Published:** 2015-09-14

**Authors:** Jo Corbett, Jade E Kirke, Thomas Eckett, Martin J Barwood, James R House

**Affiliations:** 1 Extreme Environments Laboratory, Department of Sport and Exercise Science, University of Portsmouth, Portsmouth, UK

## Introduction

There is considerable interest in reducing the ergolytic effect of heat. One approach is to reduce body-heat content through ingesting ice-slurry (IS), which provides a substantially greater heat sink benefit than cool liquids because of the enthalpy of fusion absorbed in the phase change from ice to water [1]. Many studies using IS have employed a formulation containing carbohydrate [2], which is itself ergogenic during prolonged exercise in the heat [3]. Although the separate effects of IS and carbohydrate on performance in the heat are established, it is unclear if there is an interaction when co-ingested. For example, exogenous carbohydrate oxidation is impaired with hyperthermia [4], whereas IS reduces heat stress. This study examined the separate and combined effects of IS and carbohydrate on performance, thermoregulation, substrate utilisation and thermal perception during prolonged cycling exercise in the heat.

## Methods

Using a balanced, repeated-measures design, eight physically active males (mean(SD) age 22(2) years; height 1.77(0.03) m; mass 70.29(9.23) kg; cycling peak power output (PPO) 312(51) W; VO_2max _3.31(0.59) L.min^-1^) consumed 7.5 g.kg^-1 ^body mass of either: low-sugar squash liquid (LI [~7 °C]); low-sugar squash IS (IS [~0 °C]); 6 % carbohydrate squash (LIC [~7 °C]); 6 % carbohydrate IS (ISC[~0 °C]). Thereafter, participants cycled for 75 minutes at 40 % PPO in a hot environment (30°C, 50 % rh) whilst consuming 1.25 g.kg^-1 ^body mass of the beverage every 10 minutes, before undertaking a 10 km time-trial (TT). Effects of the drink condition on dependent variables were assessed by repeated measures ANOVA with *post-hoc t*-tests and significance set at P < 0.05.

## Results

TTs were faster in LIC (18.74(1.24) min) and ISC (18.64(1.08) min) than IS (19.47(1.49) min), with a trend for LIC and ISC to be faster than LI (19.78(2.18) min). Upon commencing fixed intensity exercise rectal temperature (T_re_) was lower with IS drinks (~0.3 °C), but this difference was largely abolished before the TT; skin temperature was unaffected by beverage. Fat oxidation tended to be lower with LIC and ISC (*P *= 0.06) relative to IS at the end of the fixed intensity exercise and thermal comfort and sensation were improved with IS beverages, although RPE was unaffected.

## Discussion

Carbohydrate beverages were ergogenic following prolonged exercise in the heat and this effect was apparent with cold liquid and IS beverages, indicating that the IS did not meaningfully impair the benefits of exogenous carbohydrate supplementation. IS beverages reduced resting T_re _in the heat, but this effect did not persist following prolonged exercise, even with continued consumption. However IS drinks did provide some favourable perceptual benefits in terms of improved thermal comfort and sensation, although these did not influence performance.

## Conclusion

An IS beverage containing carbohydrate is an appropriate choice for individuals engaging in prolonged exercise in the heat, in terms of combining perceptual and performance benefits.

## References

[B1] Siegel R Laursen PB Keeping Your Cool Sports Med 2012 42 89 98 10.2165/11596870-000000000-00000 22175533

[B2] Siegel R Maté J Brearley MB Ice slurry ingestion increases core temperature capacity and running time in the heat Med Science Sports Exerc 2010 42 17 725 10.1249/MSS.0b013e3181bf257a 19952832

[B3] Davis JM Lamb DR Pate RR Carbohydrate-electrolyte drinks: effects on endurance cycling in the heat Am J Clin Nutr 1988 48 1023 1030 3421199 10.1093/ajcn/48.4.1023

[B4] Jentjens RL Wagenmakers AJ Jeukendrup AE Heat stress increases muscle glycogen use but reduces the oxidation of ingested carbohydrates during exercise J Appl Physiol 2002 92 1562 1572 10.1152/japplphysiol.00482.2001 11896023

